# Mesenchymal Stem Cell-Derived Exosomal MiRNAs Promote M2 Macrophages Polarization: Therapeutic Opportunities for Spinal Cord Injury

**DOI:** 10.3389/fnmol.2022.926928

**Published:** 2022-07-12

**Authors:** Ze-Yan Liang, Xiong-Jie Xu, Jian Rao, Zhe-Lun Yang, Chun-Hua Wang, Chun-Mei Chen

**Affiliations:** Department of Neurosurgery, Fujian Medical University Union Hospital, Fuzhou, China

**Keywords:** spinal cord injury, macrophages, mesenchymal stem cells, exosomes, microRNA

## Abstract

Spinal cord injury (SCI) is an enormous public health concern affecting approximately 250,000–500,000 people worldwide each year. It is mostly irreversible considering the limitations of currently available treatments, and its prevention and management have been the prime focus of many studies. Mesenchymal stem cell (MSC) transplantation is one of the most promising treatments for SCI. The role of MSCs in SCI has been studied extensively, and MSCs have been shown to have many limitations. Moreover, the therapeutic effects of MSCs are more likely related to paracrine effects. In SCIs, macrophages from peripheral sources differentiate into M1 macrophages, promoting inflammation and aggravating neuronal damage; however, studies have shown that MSC-derived exosomes can induce the polarization of macrophages from the M1 to the M2 phenotype, thereby promoting nerve function recovery in patients with SCI. In this review, we discussed the research progress of MSC-derived exosomal miRNAs in promoting M2 macrophage differentiation in the SCI, and introduced some exosomal miRNAs that can regulate the differentiation of M2 macrophages in non-SCI; it is hoped that the regulatory role of these exosome-derived miRNAs can be confirmed in SCI.

## Introduction

Spinal cord injury (SCI) is an enormous public health concern affecting approximately 250,000–500,000 people worldwide each year (Khorasanizadeh et al., 2019). When young people are affected, the burden of permanent nerve damage is unbearable for the patient, their families, and the health care system (Selvarajah et al., 2014). The treatment of SCI is challenging owing to the lack of a gold standard or significantly effective treatment strategy. Most post-traumatic degeneration of the nervous system is caused by multifactorial secondary damage, which includes different pathophysiological processes such as hemorrhage, ischemia, blood–brain barrier dysfunction, oxidative stress, neuronal damage, broken neural circuits, inflammatory events, scar formation, the imbalance in endogenous neural stem cells differentiation, necrosis, and apoptosis, leading to permanent functional damage (McDonald and Sadowsky, 2002; Stenudd et al., 2015; Fan et al., 2018). Because SCI is mostly irreversible using the currently available treatment methods, its prevention and management have been the primary focus of several studies. Mesenchymal stem cell (MSC) transplantation is one of the most hopeful treatments for SCI (Assinck et al., 2017). Although their roles in SCI have been studied in-depth, many shortcomings have been reported, and the results of clinical trials do not corroborate those of preclinical experiments; moreover, the therapeutic effects of MSCs may largely be related to paracrine effects (Curtis et al., 2018; Xu and Yang, 2019; Liu et al., 2021). Exosomes are significant mediators of cell-to-cell interaction regulation and take part in many pathophysiological processes. The therapeutic potentials of exosomes in SCIs have recently been receiving increasing attention. MSC-derived exosomes may participate in cell regulation by transporting proteins, lipids, and RNA (Wang et al., 2018; Ren et al., 2020).

Macrophages present in the peripheral circulation, including phenotypes such as M1 (classically activated) and M2 (alternatively activated), are a key effector in the inflammatory response after SCI (Gordon, 2003; Mantovani et al., 2004; Beck et al., 2010). M1 macrophages promote secondary inflammation after SCI, whereas M2 macrophages exert anti-inflammatory effects and promote axon regeneration (David and Kroner, 2011). In SCI, macrophages of peripheral origin mainly differentiate into M1-type macrophages (Kigerl et al., 2009; Zhou et al., 2014). However, studies have shown that MSC-derived exosomes can induce the polarization of macrophages from the M1 to the M2 phenotype, thereby promoting neurological function recovery in patients with SCIs (Sun et al., 2018; Liu et al., 2021). Herein, we review the mechanism *via* which MSC-derived exosomal miRNAs promote M2 macrophage differentiation, providing a new target for SCI treatment in the future.

## Macrophage Plasticity

A typical, continuous inflammatory cascade begins after SCI. Within 24 h after SCI, neutrophils are recruited from the circulation, activating central glial cells (astrocytes and microglia; Davalos et al., 2005; Pekny and Nilsson, 2005). Soon thereafter (2–3 days after SCI), blood mononuclear cells migrate to the injury site and differentiate into macrophages; these macrophages cluster in the center of the injured spinal cord and coordinate inflammatory responses (Hawthorne and Popovich, 2011). Recent researches have suggested that the macrophage is highly plastic and could convert their phenotype and function by virtue of variations in the microenvironment (El Kasmi and Stenmark, 2015; Shapouri-Moghaddam et al., 2018). IFNγ and lipopolysaccharide (LPS)-induced STAT1 signaling and NF-κB signaling stimulate macrophages to express the classic M1 phenotype (Orecchioni et al., 2019). M1 macrophages generate proinflammatory cytokines (TNF-α and IL-1), reactive oxygen species, and nitric oxide, leading to tissue inflammation and damage (Porta et al., 2015). In contrast, IL-4 binds IL-4Ra and activates STAT6, activating key transcription factors for M2-like polarization, including PPARδ, KLF4, and PPARγ (Odegaard et al., 2008; Porta et al., 2015). A combination of IL-10 and IL-10R activates the IL-10/JAK1/STAT3 cascade; the phosphorylated STAT3 homodimer then translocates to the nucleus within a few seconds to activate the expression of target genes such as Bcl3, thereby inhibiting the inflammatory response and promoting the expression of M2 macrophage-related cytokines (Hutchins et al., 2013). The epigenetic landscape, transcription factors, and microRNA networks are the basis of the adaptability of macrophages to different environmental cues (Locati et al., 2020).

## MSC-Derived Exosomes

**Figure 1:**
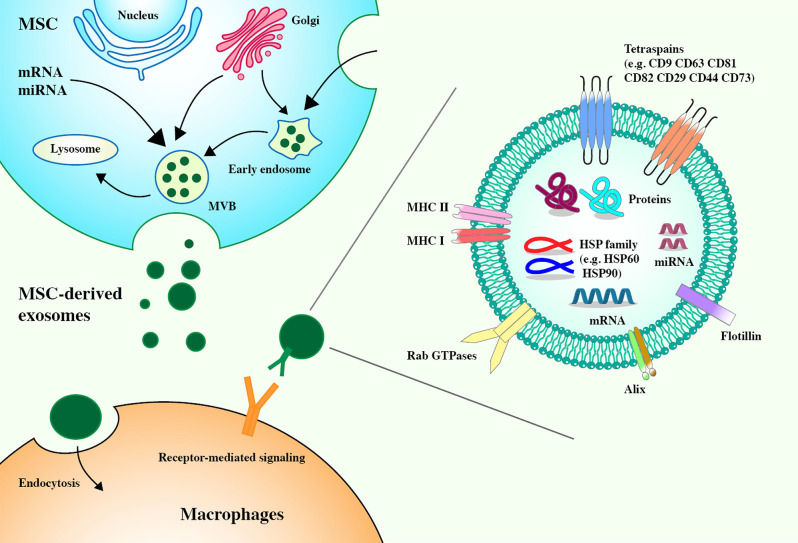
Mesenchymal stem cell-derived (MSC-derived) exosomes express tetraspanins (CD82, CD81, CD63, and CD9), heat shock proteins (HSP60, HSP70, and HSP90), membrane transport, and fusion proteins (GTPase, annexin, and flotillin), ALG-2 interacting protein X (Alix), and adhesion molecules (CD29, CD44, and CD73). Exosomes derived from MSCs carry a complex cargo, including nucleic acids, and proteins. Biogenesis of MSC-derived exosomes. The biogenesis of exosomes includes the formation of early endosomes through the invagination of the plasma membrane, the formation of late endosomes through the selection of cargo, and the formation of multivesicular bodies (MVBs) from late endosomes. MVBs contain intraluminal vesicles. The fusion between MVBs and the plasma membrane results in the release of exosomes. The ways for exosomes to enter recipient cells are receptor-mediated entry and endocytosis.

Exosomes are extracellular vesicles (EVs) that are released into the extracellular environment. They can act very close to or far away from the parent cells. They have a diameter of 40–100 nm and are secreted by almost all cell types. They are widely present in body fluids (Raposo and Stoorvogel, 2013; Colombo et al., 2014; Prada and Meldolesi, 2016). Existing clinical studies have shown that most of the immunomodulatory effects of MSCs can be attributed to the immunomodulatory properties of MSC-derived exosomes, which secrete more exosomes than other cells (Yu et al., 2014; Harrell et al., 2019). MSC-derived exosomes contain membrane transport and fusion proteins (GTPase, annexin, and flotillin), transmembrane proteins (CD9, CD63, CD81, and CD82), heat-shock proteins (HSP60, HSP70, and HSP90), ALG-2-interacting protein X (Alix), and adhesion molecules (CD29, CD44, and CD73) (Schorey and Bhatnagar, 2008; Colombo et al., 2014; Yu et al., 2014). MSC-derived exosomes carry abundant MSC-derived biologically active molecules, including messenger RNAs (mRNAs), microRNAs (miRNAs), cytokines, chemokines, and immunomodulatory and growth factors, which can regulate the phenotype and function of immune cells (Harrell et al., 2019; Yao et al., 2021; Figure 1). After reaching the target cell, the MSC–EV membrane can fuse with the target cell membrane to directly release its RNA or protein or the MSC–EV membrane protein can bind the target cell membrane protein to activate a series of signaling pathways (Kusuma et al., 2018). Compared with MSCs, MSC-derived exosomes can be easily separated and stored and are almost free from ethical restrictions (Gimona et al., 2017). As a medical product, MSC–EVs have theoretical advantages over complete MSCs and, in the future, may take precedence over whole cells in regenerative medicine (Rani et al., 2015).

## MSC-Derived Exosomal MicroRNAs Promote M2 Macrophage Polarization

Existing evidence has shown that after SCI, in addition to the resident microglia of the central nervous system, MSC-derived exosomal MicroRNAs are involved in inflammation, cell proliferation, differentiation, and tissue remodeling, and some of them are mediated by the differentiation of circulating monocytes into macrophages (Davalos et al., 2005; Beck et al., 2010; Li and Barres, 2018). The injured tissue releases related cytokines and chemokines into the circulation and guides circulating monocytes to the injured part; differentiation into macrophages then takes place, aggravating SCI (Wang et al., 2015). Although microglia are called “central nervous system macrophages” and share many functions with their peripheral macrophage cousins, infiltrating macrophages and resident microglia have different functions; the difference lies in protein expression, phagocytosis, and response to injury (Durafourt et al., 2012; Hu et al., 2015). The origin of macrophages in SCI and their contribution to spinal cord pathology refer to related reviews (Milich et al., 2019).

MSC-derived exosomes often contain abundant nucleic acids, which play a vital role in altering the fate of recipient cells. Among these, microRNAs (miRNAs) are the most studied. Most infiltrating macrophages in the injured spinal cord are M1 macrophages, and only a few transient M2 macrophages exist (Kigerl et al., 2009); further, due to the superiority of the number of M1 macrophages, SCI may be exacerbated (David and Kroner, 2011). Many studies have reported that MSC-derived exosomes carrying specific miRNAs can promote the polarization of M2 macrophages (Song et al., 2017; Yao et al., 2021).

## Biogenesis of MiRNAs

MiRNAs are short noncoding single-stranded RNAs comprising approximately 22 nucleotides; they regulate post-transcriptional gene expression by directing target mRNA cleavage or by inhibiting translation (Ambros et al., 2003). In the cell nucleus, miRNA is transcribed by RNA polymerase II (Pol II) into monocistronic or polycistronic, long, primary precursor transcript miRNA. Then, it encounters the RNase III endonuclease Drosha and the double-stranded RNA-binding domain protein DGCR8/Pasha and is cleaved into an approximately 70-nt precursor miRNA (Lee et al., 2003; Winter et al., 2009). Subsequently, the precursor miRNA is transported to the cytoplasm by exportin-5, and the second RNase III endonuclease Dicer is cleaved and processed into approximately 21-nt duplexes together with the double-stranded RNA-binding domain protein TRBP/Loquacious (Bohnsack et al., 2004; Kim, 2004; Ji, 2008). Then, one strand of the short double-stranded RNA is integrated into the RNA-induced silencing complex, and the complementary strand is subsequently degraded (Kawamata and Tomari, 2010; Newman and Hammond, 2010). miRNAs regulate gene expression after transcription and interact with the 3’untranslated region of the target mRNA to induce mRNA degradation and inhibit translation (Bushati and Cohen, 2007). miRNAs participate in various cellular processes, including cell metabolism, cell differentiation, cell proliferation, cell death, and immune responses in various physiological and pathological processes (Gauthier and Wollheim, 2006; Matsubara et al., 2007). More than one-third of all human genes are regulated by miRNAs (Esquela-Kerscher and Slack, 2006). miRNAs can be secreted into the extracellular fluid and transported to target cells through vesicles such as exosomes or by binding to proteins (including Argonautes; O’Brien et al., 2018).

## MicroRNA Function in M2 Macrophage Polarization

The complex gene network and the signal cascade of macrophage polarization are regulated by multilayer gene expression. Transcription factors can define the identity of macrophages and control their number and function by inducing and maintaining specific transcription programs (Molawi and Sieweke, 2013). Genome-wide studies related to transcription and epigenetics have determined that differences in miRNAs strongly influence the type and duration of macrophage-mediated inflammation (Locati et al., 2020).

## Exosomal miRNAs Promote M2 Macrophages Polarization in SCI

**Figure 2:**
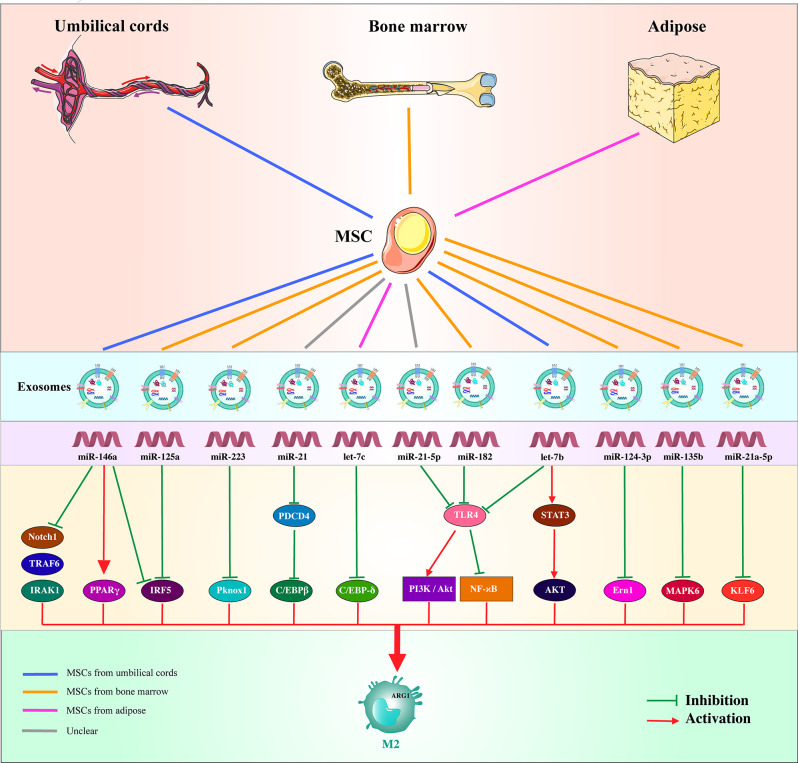
Diagram illustrates MSC-derived exosomal miRNAs that induce alternative activation of macrophages and their corresponding targets.

The key role of miRNAs in driving the development and maturation of immune cells has been confirmed. Several studies have described complex regulatory networks between miRNAs and key transcriptional regulators that control the phenotype and function of macrophages (Locati et al., 2020). At present, miRNAs present in exosomes secreted by various MSCs have been confirmed to induce and promote the polarization of M2 macrophages. In miR-146a-overexpressing macrophages, a decrease in M1 phenotypic marker (iNOS and TNF-α) and an increase in M2 marker gene (Arg1 and IL-10) levels were observed. Moreover, the source of exosomal miR-146a can regulate the polarization of macrophages by targeting PPARγ and silencing multiple inflammatory targets such as Notch1, IRAK1, TRAF6, and IRF5 and inhibiting NF-κB-driven TNF-α expression in macrophages (Li et al., 2015; Huang et al., 2016; Song et al., 2017). The endoplasmic reticulum (ER) is considered a powerful controller of inflammatory signals and immune cell responses to various stimuli (Martinon et al., 2010; Cho et al., 2013). ER stress is a pathological result of SCI (Wang et al., 2017). Endoplasmic reticulum-to-nucleus signal 1 (Ern1) is an ER-anchored kinase that activates the transcription factor XBP1, forming the most conserved signal branch of ER stress (Ron and Walter, 2007). Ern1 activation can promote TLR-mediated macrophages to produce proinflammatory cytokines, which are potential therapeutic targets for inflammatory diseases (Qiu et al., 2013). A rat model of spinal cord ischemia-reperfusion injury was subjected to exosomes derived from MSCs containing miR-124-3p; miR-124-3p was found to protect the spinal cord by inhibiting the expression of Ern1 polarization of M2 macrophages at the injured site, improving the neuromotor function of rats (Li et al., 2020). In addition, in SCI, miR-125a in exosomes secreted by MSCs promotes M2 macrophage polarization by targeting and downregulating IRF5 expression (Chang et al., 2021; Figure 2).

## Exosomal miRNAs Promote M2 Macrophages Polarization in Non-SCI

At present, there are limited reports that exosome-derived miRNAs promote the conversion of macrophages to the M2 type. However, in other animal models of diseases, a variety of exosome-derived miRNAs have been shown to promote the polarization of M2 macrophages. MiR-223 can promote the conversion of macrophages to the M2 phenotype. miR-223 targets and inhibits C/EBPβ to limit the release of LPS-dependent IL-1β and IL-6 cytokines, thereby weakening the M1 macrophage proinflammatory activity of cells and enhancing alternative anti-inflammatory responses (Zhou et al., 2015). The miR-223 in exosomes also directly targets Pknox1, inhibiting its expression and regulating macrophage polarization (He et al., 2019). Programmed cell death protein 4 (PDCD4) can be combined with C/EBPβ to affect macrophages instead of activating signaling pathways. The miR-21 carried by MSC-derived exosomes can downregulate PDCD4, creating a positive effect to promote the M2 phenotype of macrophages (Zhong et al., 2014; Yao et al., 2021). By targeting C/EBP-δ, let-7c expression can impair the release of M1-related genes (iNOs and IL-12) and increase the level of M2 markers (FR-β; Hao et al., 2021).

Toll-like receptor 4 (TLR4) is a member of a pattern recognition receptor subfamily that can recognize invading pathogens and endogenous harmful stimuli to induce innate and adaptive immune responses (Swanson et al., 2020). TLR4 forms a complex with coreceptor myeloid differentiation protein 2, which recognizes LPS and binds it to trigger a signal cascade, such as the NF-κB pathway, to produce proinflammatory cytokines (TNFα, IL-1β, IL-6, and IL-12; Lu et al., 2008). MiR-21-5p, miR-182, let-7b, and other miRNAs derived from exosomes secreted by MSCs target the expression of TLR4 to mediate macrophage polarization (Ti et al., 2015; Zhao et al., 2019; Shen and He, 2021). MiR-182 enhances the PI3K/Akt signaling pathway by inhibiting TLR4, which plays an important role in the transformation of anti-inflammatory M2 macrophages (Zhao et al., 2019). Let-7b is a member of the let-7 family and has a different expression pattern in inflamed tissue and healthy tissue (Guo et al., 2015). The mutual inhibition crosstalk between NF-κB and STAT3 can regulate the M1/M2 balance and coordinate the response to different microenvironments; NF-κB activation can induce an inflammatory M1 phenotype, whereas STAT3 activation can promote anti-inflammatory M2 conversion (Gao et al., 2014). Let-7b carried by exosomes is transferred to M1 type macrophages, which inhibit NF-κB and activate STAT3 by downregulating the expression of TLR4 (Ti et al., 2015). STAT3 can induce AKT activation, which affects immune homeostasis and promotes M2-like phenotype-related effector gene (IL-10) expression, thereby regulating macrophage polarization (Ke et al., 2012; Hutchins et al., 2013).

Mitogen-activated protein kinase (MAPK) is a type of serine-threonine protein kinase that can transform extracellular stimuli into a wide range of cellular responses and participate in the coordination of basic processes such as cell–gene expression, mitosis, metabolism, survival, apoptosis, and differentiation (Raman et al., 2007). MAPK6/ERK3 is an unconventional MAPK, lacking the typical Thr-X-Tyr motif and containing a Ser-Glu-Gly motif in the activation loop of the classic MAPK kinase domain (Cargnello and Roux, 2011). MAPK6 is clearly involved in myocardial ischemia-reperfusion injury and promotes hypoxia-induced cardiomyocyte damage (Luo et al., 2020). Furthermore, MSC-derived exosomal miR-135b can downregulate MAPK6 to induce macrophages to polarize to the M2 type (Wang and Xu, 2021). However, the specific signaling pathway *via* which MAPK6 participates in macrophage polarization requires further study. Kruppel-like factor 6 (KLF6) is a member of the zinc finger transcription factor family, which regulates key cellular processes such as cell development, cell differentiation, cell proliferation, and programmed cell death (Kim et al., 2020). Studies have shown that KLF6 can increase the expression of proinflammatory genes by reducing the expression and activity of PPARγ while cooperating with NF-κB to promote the polarization of M1 macrophages (Date et al., 2014; Kim et al., 2020). MSC-derived exosomal miR-21a-5pcan target the downregulation of KLF6 expression, weaken a wide range of proinflammatory gene programs, and induce the polarization of M2 macrophages (Ma et al., 2021; Figure 2).

## Future Directions

MSC transplantation seems to be an ideal and highly anticipated treatment for SCI. However, MSC transplantation poses potential risks. MSC transplantation causes chromosomal abnormalities in early passages and leads to tumors (Jeong et al., 2011). In addition, MSCs did not directly differentiate into neuronal cells to promote spinal cord repair, as expected. The therapeutic effect of MSC transplantation is attributed to its paracrine activity. MSCs secrete exosomes containing high levels of specific miRNAs by transfecting specific miRNA plasmids in advance; this gives them the advantage of size (nanometers), allowing them to pass through the blood–spinal cord barrier and play an important role in nervous system repair.

It is now very clear that macrophages play a key role in the progression of SCI as well as post-repair damage. The type of macrophages affects the progression and repair of SCIs and has a profound impact on the overall neuronal function and SCI results. MSC-derived exosomes are effective in the treatment of SCI; crucial miRNAs participate in and regulate the polarization of macrophages. This article reviewed the existing related studies and found that MSC-derived exosomal miRNAs downregulate the expression of IRAK1, TRAF6, IRF5, C/EBPβ, C/EBP-δ, TLR4, PDCD4, Enr1, MAPK6, and KLF6 by targeting and inhibiting the macromolecule expression, promoting the polarization of M2 macrophages. These mechanisms will guide future research related to SCI treatment and promote the search for new therapeutic targets.

There remain several challenges before clinical trials on exosomal miRNAs for SCIs can commence. At first, there are also cytokines, chemokines, immune regulators, and growth factors in exosomes, which may be involved in the regulation of immune cell phenotype; however, current studies on miRNAs regulating macrophage polarization have not explained whether other components are involved in the regulation of macrophage polarization. Then, how to increase the content of target miRNAs in exosomes needs to be further explored. At present, there is a lack of research on how exosomes are enriched to target regions after entering the body. In addition, in the study of exosome treatment for SCI, the time of infusion of exosomes has not been unified. Nevertheless, the regulation of macrophage polarization by most miRNAs has not been verified in animal models of SCI. Further studies are needed to verify whether these miRNAs can also regulate the polarization of macrophages to M2 in the SCI microenvironment (Wang and Xu, 2021; Yao et al., 2021).

In general, the treatment of SCI is challenging, and there is no effective strategy to repair lost nerve function. The secondary injury process after SCI is very complicated and is a result of various factors that delay the development of treatment methods for complete recovery after injury. MSC-derived exosomes may bring new therapeutic hope to SCI patients. However, it still needs more effort from scholars around the world to explore to realize this vision.

## Author Contributions

C-MC and Z-YL developed the concept of the manuscript. Z-YL and C-HW conducted the analysis of the literature. Z-YL, X-JX, JR, and Z-LY composed and edited the manuscript text and figures. All authors contributed to the article and approved the submitted version.

## Conflict of Interest

The authors declare that the research was conducted in the absence of any commercial or financial relationships that could be construed as a potential conflict of interest.

## Publisher’s Note

All claims expressed in this article are solely those of the authors and do not necessarily represent those of their affiliated organizations, or those of the publisher, the editors and the reviewers. Any product that may be evaluated in this article, or claim that may be made by its manufacturer, is not guaranteed or endorsed by the publisher.
